# Spatial priority for COVID-19 vaccine rollout against limited supply

**DOI:** 10.1016/j.heliyon.2021.e08419

**Published:** 2021-11-17

**Authors:** Showmitra Kumar Sarkar, Md. Manjur Morshed

**Affiliations:** Department of Urban and Regional Planning, Khulna University of Engineering & Technology (KUET), Khulna, 9203, Bangladesh

**Keywords:** Pandemic, Demography, Economy, Vulnerable groups, GIS, Bangladesh

## Abstract

The COVID-19 vaccines are limited in supply which requires vaccination by priority. This study proposes a spatial priority-based vaccine rollout strategy for Bangladesh. Demographic, economic and vulnerability, and spatial connectivity – these four types of factors are considered for identifying the spatial priority. The spatial priority is calculated and mapped using a GIS-based analytic hierarchy process. Our findings suggest that both demographic and economic factors are keys to the spatial priority of vaccine rollout. Secondly, spatial connectivity is an essential component for defining spatial priority due to the transmissibility of COVID-19. A total of 12 out of 64 districts were found high-priority followed by 22 medium-priorities for vaccine rollout. The proposed strategy by no means suggests ending mass vaccination by descending age groups but an alternative against limited vaccine supply. The spatial priority of the vaccine rollout strategy proposed in this study might help to curb down COVID-19 transmission and to keep the economy moving. The inclusion of granular data and contextual factors can significantly improve the spatial priority identification which can have wider applications for other infectious and transmittable diseases and beyond.

## 1 Introduction

Vaccination against COVID-19 is a precondition for normalizing daily life and opening up economic activities. The rising number of COVID-19 cases globally – potentially the third wave, mostly by the Delta variant – from March 2021 onwards is a renewed concern for the world, especially in the worst affected countries like the UK, the USA, Indonesia, India and Brazil. The public sentiment is against an all-out lockdown as the pandemic has caused a 3.5 percent contraction of the global economy in 2020 [1]. The social costs of the pandemic are understood yet have mostly remained unaccounted for. Curbing the pandemic through vaccination is a challenge because the availability of vaccines varies across countries, especially the low-income countries are lagging considerably [1, 2].

Globally, the number of available vaccines is in short supply, and vaccine rollout is prioritized in every country. The Advisory Committee on Immunization Practices (ACIP) in the US recommended vaccine rollout with the goal in mind “to decrease death and serious illness due to COVID-19 as much as possible, to maintain the functioning of society and to reduce the extra burden on people already facing disparities.” Health care personnel and care home residents are the first to receive the vaccination. Frontline workers and people over the age of 75 received vaccination in the second phase followed by descending age groups in the third phase [3]. The UK approach to vaccine rollout is similar to the USA with added categories of ethnic minorities, obese and deprived communities [4].

The sheer scarcity of vaccines has led to a vaccine hoarding situation where well-to-do countries have invested heavily in vaccine procurement. The poorer nations are expected to receive the lion's share of their vaccines from the COVAX initiative, a coalition among the Coalition for Epidemic Preparedness Innovations (CEPI), the Vaccine Alliance (GAVI) and the World Health Organization (WHO). As of 21 July 2021, the COVAX program has delivered over 138 million COVID-19 vaccines [5], which is far short of the global demand. As a result, most countries i.e., Bangladesh are relying on national procurement and partisan treaty. The scarcity of vaccines has led some nations to budget vaccination according to their national priority. For example, Indonesia has prioritized the working-age people over the older ones, a move criticized as a very economic one.^1^

**Figure 1:**
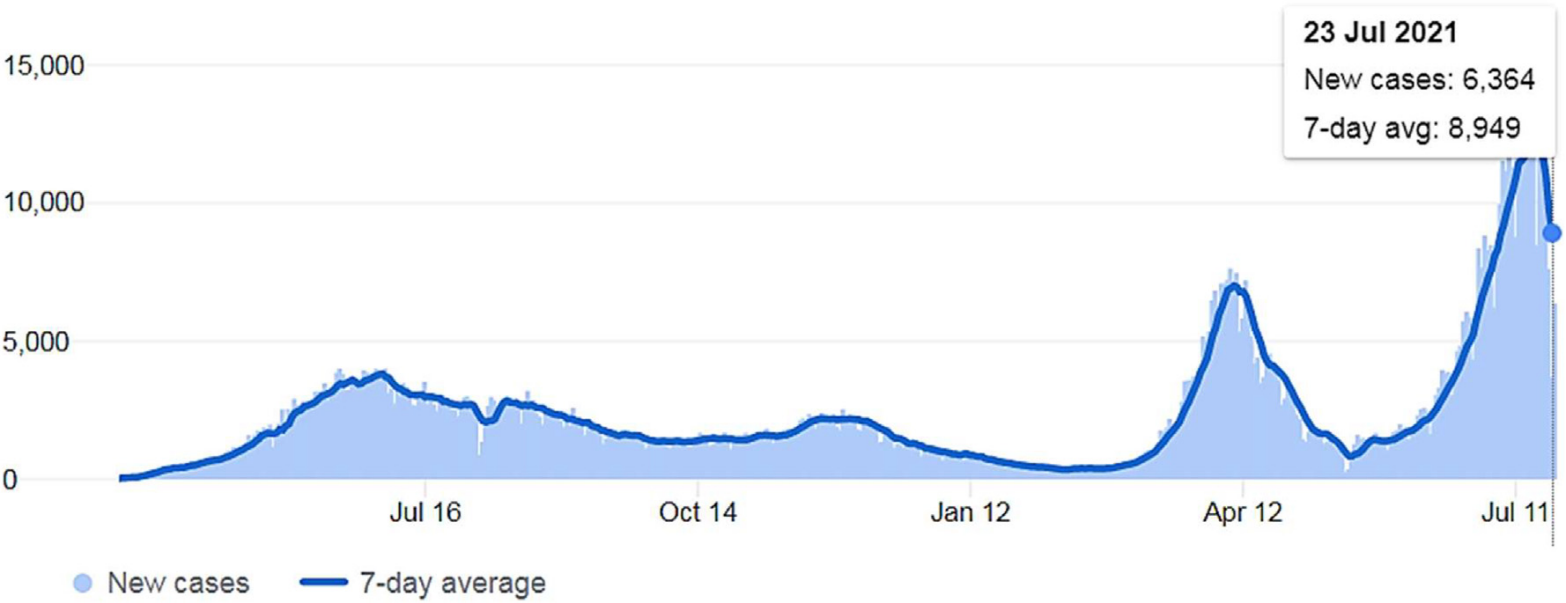
COVID-19 case trend in Bangladesh [7].

Bangladesh, which is the focus of this study, has a similar experience with the global trend of the third wave (Figure 1) [6]. The first phase of vaccination prioritized the healthcare service providers and frontline workers, and population over the age of 40. So far, the country has received 10.3 million out of 30 million doses of Oxford-AstraZeneca COVID-19 vaccine produced by the Serum Institute of India under a tripartite agreement. The rest of the vaccine delivery hit rock-bottom as India prioritized national demand over export due to the worsening coronavirus condition. As of 24 July 2021, the country received 100,620 doses of Pfizer-BioNTech and 5.5 million Moderna vaccines through the COVAX [5] 2. Additionally, Bangladesh has purchased 15 million doses of Sinopharm vaccines, of which 2 million are already been delivered in an addition to 1.1 million as a gift.^3^ However, the number of vaccines is far too short for mass vaccination of the country's more than 165 million population. Bangladesh ranks 29^th^ in the world in vaccination per 100 people, only 2.6% are fully and 1.06% are partially vaccinated [7]. The country is forced to prioritize the vaccine rollout against faltering supply and limited stock.

The objective of this research is to propose a spatial priority of the COVID-19 vaccine rollout strategy for Bangladesh. In doing so, we first consider the spatial distributions of COVID-19 cases by districts, which is highly skewed toward cities. In addition, we consider demographic, economic, vulnerable groups and spatial factors as keys for vaccination priority. So far the Bangladesh government's mass-vaccination policy has resulted in thinly distributing the limited vaccine supply. We hypothesize that vaccine rollout needs to be spatially prioritized based on the aforementioned factors.

Numerous studies are in circulation concerning the causes and consequences of COVID-19. One set of studies link COVID-19 cases with urbanization [8, 9, 10]. Cities and urban areas are more susceptible to COVID-19 than rural areas due to four key reasons: high density and connectivity, crowded living, and exposed occupation [11].^4^ However, such urban concentration of the virus is challenged as the spread is shifting towards rural areas [12] 5. Another set of studies link pandemic transmission vulnerability to the urban poor and marginalized communities [13, 14]. A few studies recommend better knowledge on pandemic adaptive urban planning as pandemic frequency is expected to be on the rise in the future [15, 16]. However, there is very little research on how vaccination is a key to pandemic emergency response against the backdrop of a limited supply; this paper expects to fill in this research gap.

An analytical hierarchy process (AHP) is applied to weigh the multiple factors to measure their respective priority. Spatial information on priority areas is calculated and mapped using geographic information system (GIS). Against this background, the second section of the paper highlights the vaccine rollout priorities and the third section explains the method and data. The fourth section is results followed by discussion and conclusion in the fifth and sixth sections, respectively.

## 2 Vaccine rationing against limited supply

Suppressive interventions including lockdown, contact tracing and quarantine, and social distancing are short-term measures against the COVID-19 pandemic [17]. A quick rebound of transmission if suppressive interventions are relaxed [18], the possibility of simultaneous outbreaks [19], and the spread of local variants, such as the Delta variant – these factors leave no alternative but to mass-vaccination as a priority to end the pandemic [17, 20]. An extraordinary amount of discussion has been directed to equitable distribution of the already approved vaccines, which is in line with WHO's slogan “no one is safe until everyone is safe” (see for example, [21, 22, 23, 24]). Not to curtail the success of the COVAX program, the global vaccine supply has been more or less nationalized where the rich nations outstrip the low-income countries by a large margin in vaccination rate (see [25]).

Secondly, from the economic point of view, unequal distribution of the COVID-19 vaccines could result in loss of the global economy up to US $1.2 trillion per year in GDP [26]. Suppressing measures against the pandemic transmission has costed the low-income countries dearly, and the lower socio-economic groups are the hardest hit [27]. Easing social distancing restrictions and opening up economic activities despite pandemic rebound is a fact for developing and emerging economies. Thirdly, the spatial distribution of COVID-19 cases has been a key consideration for the fight against the pandemic. Cities, in particular, have the highest concentration of COVID-19 cases due to density [28]. Urban areas act as transmission nodes for virus spread. Additionally, cities are engines of economic and administrative activities. Therefore, cities are a priority for vaccine rollout from the spatial perspective.

Drawing on the WHO's Pandemic Influenza Risk Management guidance, vaccination priority must be based on ethical consideration and local risk assessment [29]. At the same time, the WHO guidelines for managing scarce COVID-19 prevention and care resources recommend consideration of the local context and extent of scarcity [2, 17, 30]. In contrast to the Bangladesh government's descending age category except for the selected groups i.e., public university and medical students, we prioritize vaccine rollout in Bangladesh based on four criteria:

• demographic factors – the number of confirmed COVID-19 cases, age bracket (40+), and population distribution and density are considered under this criteria.
• economic factors – Bangladesh has been on on-and-off lockdown, restricted mobility, and social distancing since March 2020, causing an enormous economic impact. The service sector employment of the country is 40.38 percent of the total, followed by the agricultural (38.3%) and industrial (21.32) sectors ([31]; see also [32]). The service and industrial sectors have been heavily impacted by the pandemic. In response, the government has issued several stimulus packages to support the service and industrial sectors [11]. Therefore, easing restrictions and opening up economic activities are priorities for vaccine rollout.
• high risk and vulnerable groups – this includes essential health and service providers i.e., cleaners and bankers, and population over the age of 40, the poor, and people with disabilities. The essential health workers and service providers are excluded from the vaccine roll-out priority as they have been vaccinated in the first phase.
• spatiality – the COVID-19 dashboard in Bangladesh provides data at the district level, and vaccine rollout has been through the district health administration. We, therefore, are unable to perform granular scale vaccination priority and resorted to district-level analysis.

A number of researches highlight the environmental factors of the pandemic [33, 34]. However, we do not incorporate environmental factors in our analysis due to a lack of data at the district level.

## 3 Method and data

### 3.1 Description of the study area

**Figure 2:**
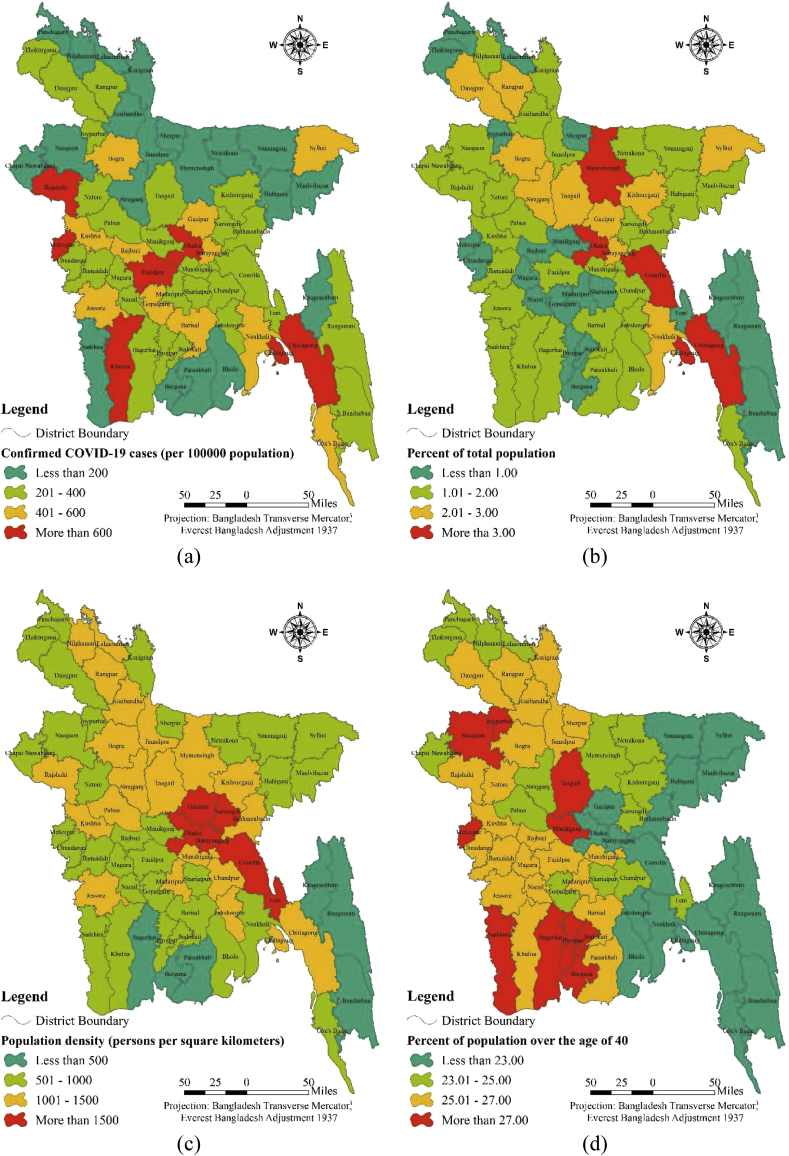

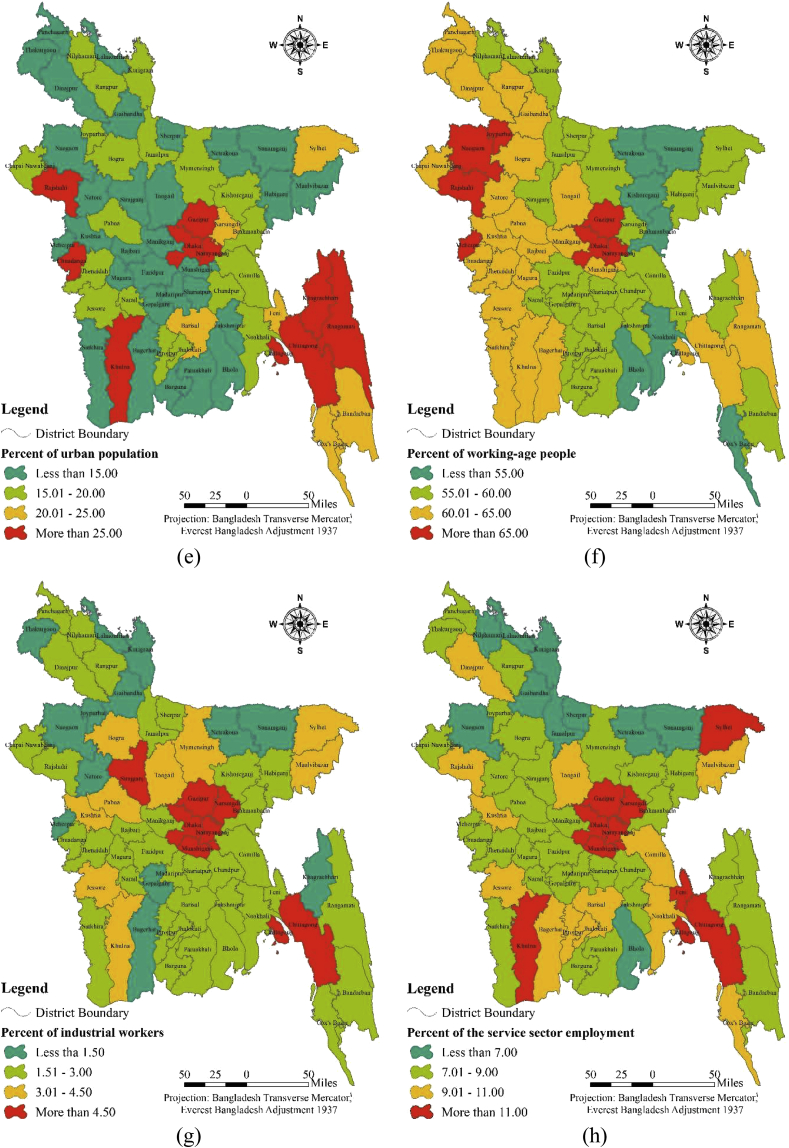

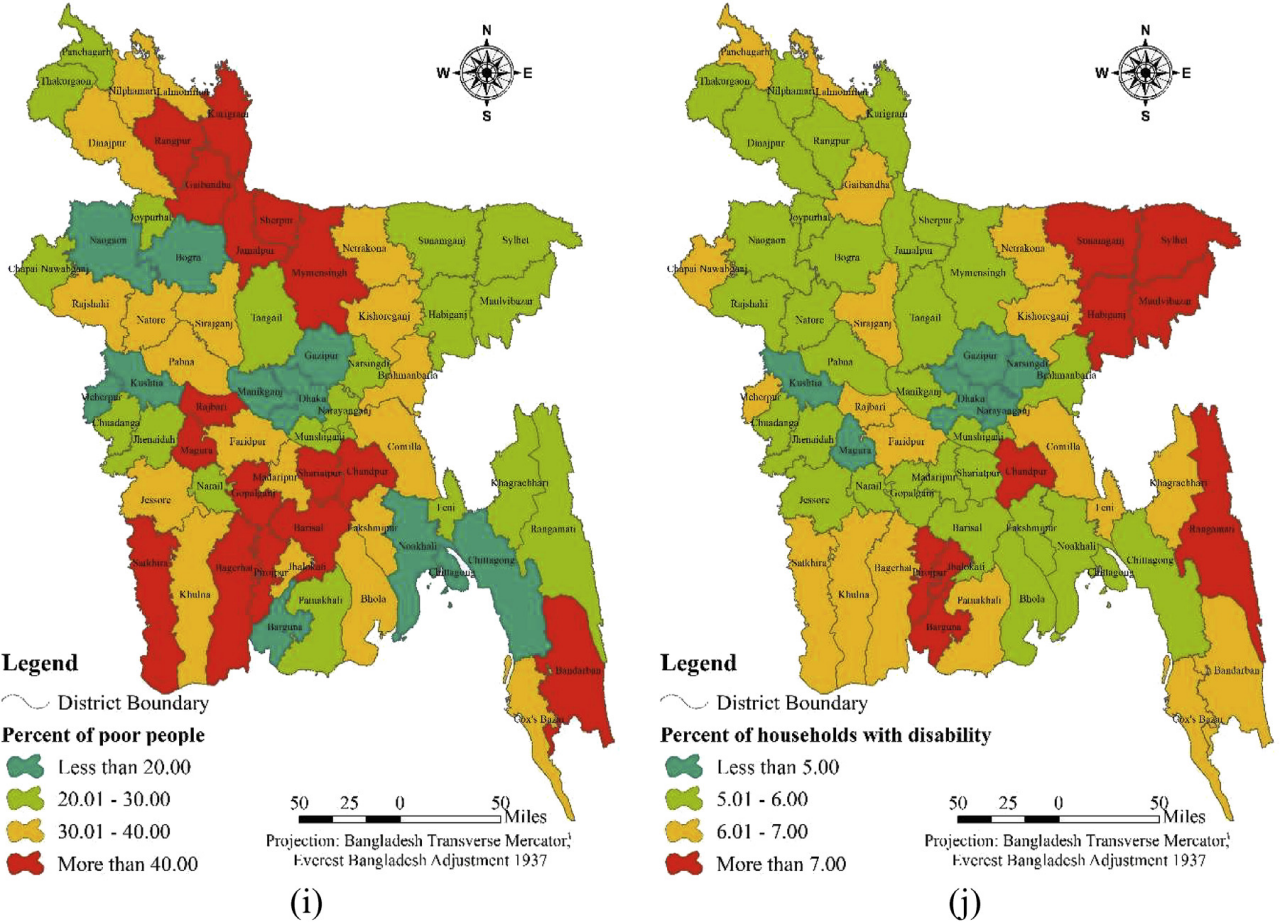
Spatial priority of the factors: (a) confirmed COVID-19 cases (per 100000 population), (b) percent of total population, (c) population density (persons per square kilometers), (d) percent of population over the age of 40, (e) percent of urban population, (f) percent of working-age people, (g) percent of industrial workers, (h) percent of the service sector employment, (i) percent of poor people, (j) percent of households with disability.

Bangladesh is one of the fastest-growing economies in the world [35]. The country has a population of over 165 million and consists of 64 administrative districts. On 08 March 2020, three COVID-19 positive patients were confirmed for the first time [36]. As of 24 July 2021, a total of 1,153,344 confirmed COVID-19 cases and 19,046 deaths were officially reported (Figure 1). Figure 2 (a) illustrates the district-wise distribution of COVID-19 confirmed cases. As of 24 July 2021, a total of 6,033,439 first doses and 4,289,518 second doses of COVID-19 vaccines have been administered nationwide [7].

### 3.2 GIS-based priority mapping

The research applied a knowledge-based approach (i.e., multi-criteria) to assess the vaccine rollout priority of districts in Bangladesh. GIS-based priority mapping by using the analytical hierarchy process (AHP) is a widely used method in the field of multi-criteria decisions [37], which uses mathematical and psychological principles for investigating multi-criteria decisions [38]. Numerous studies have addressed COVID-19 related issues using the GIS-based AHP method (for example, susceptibility/risk mapping [39, 40, 41], and prioritization of activities and facilities [42]). However, literature on GIS-based AHP for vaccine rollout is scarce. This research utilized a GIS-based AHP method for priority mapping for COVID-19 vaccine rollout. For an initial investigation, each factor was randomly classified into four sub-factors and the factor maps were prepared. The hierarchy of factors using pairwise comparisons helps to discover and correct logical inconsistencies in AHP [43, 44]. Pairwise comparisons of two factors or two sub-factors under a factor were conducted. Weights (i.e., equal importance, more importance, and less importance) [45, 46] were given for differentiating two factors or two sub-factors under a factor. A 9-point continuous scale (i.e., 1/9, 1/8, 1/7, 1/6, 1/5, 1/4, 1/3, 1/2, 1, 2, 3, 4, 5, 6, 7, 8, 9) was used for weight rating [47, 48]. The given weight rating was used for calculating the eigenvalues and consistency ratio (CR). CR was calculated using the following Eq. (1) [49, 50].

(1) $$CR=\frac{CI}{RI}$$

where, RI = mean consistency index; CI = consistency index.

The consistency index (CI) was calculated using the following Eq. (2).

(2) $$CI=\frac{y_{max}-N}{\left(N-1\right)}$$

where, y_max_ = the largest eigenvalue; N = order of the comparison matrix.

The CR values < 10% was accepted [51]. The factor maps were reclassified based on the calculated weights of sub-factors. Finally, priority maps were prepared using GIS-based weighted overly using reclassified factor maps and weights of factors. The following Eq. (3) was used for priority level calculation.

(3) $$Priority\;level=\overset{n}{\underset{j=1}{\sum }}Wj*w_{ij}$$

where, W_j_ = weight of causative factor j; w_ij_ = weighted value of class i of causative factor j.

The 64 districts were classified into three classes based on priority level based on the Jenks’ natural break method: (1) high priority; (2) medium priority; and (3) low priority.

All districts of Bangladesh are spatially inter-connected and influence each other in COVID-19 transmission. To address the influence of other districts on the priority level of one district, we integrated the distance function with the priority map calculated from the GIS-based AHP. We proposed a relationship between priority level based on district weight from AHP and road distance between districts (in Table 1). Finally, the spatial priority of vaccine rollout was calculated using the priority level and road distance function. The 64 districts were further reclassified into three classes based on spatial priority level using the Jenks’ natural break method: (1) high priority; (2) medium priority; and (3) low priority.

**Table 1:** Proposed relationship between priority level and road distance.

Percent of influence of a district on others	Distance between districts (km)
50%	≤100
25%	101–200
12.50%	201–300
6.25%	301–400
3.13%	401–500
1.56%	501–600
0.78%	601–700

### 3.3 Data

Four key areas are considered for vaccine roll-out priority: demographic factors, economic factors, vulnerability, and spatiality. Five demographic (i.e., number of confirmed COVID cases, percent of the population, population density, percent of the urban people, percent of the population over the age of 40), three economic (i.e., percent of the working-age people, percent of the industrial workers, percent of the service sector employment), and two vulnerability (i.e., percent of poor people, percent of households with disability) factors were used for multi-criteria based priority ranking. Finally, spatial priority is calculated based on road distance between districts. This research is based on secondary data that are mentioned in Table 2.

**Table 2:** Description of data.

Data with units	Mean	Minimum	Maximum	Standard deviation	Source with year
Confirmed COVID-19 cases (per 100000 population)	339.50	84.81	2965.84	369.73	Institute of Epidemiology Disease Control and Research (2021)
Percent of total population	1.56	0.27	8.36	1.20	Bangladesh Bureau of Statistics (2011 Census)
Population density (persons per square kilometres)	1107.69	86.00	8111.00	1030.07	
Percent of population over the age of 40	24.61	17.07	28.90	2.52	
Percent of urban population	17.84	8.80	77.10	10.15	
Percent of working-age people	59.96	52.81	70.70	3.86	
Percent of industrial workers	3.12	0.64	22.78	3.46	
Percent of the service sector employment	9.23	5.49	27.69	3.29	
Percent of poor people	32.25	3.59	63.72	11.95	
Percent of households with disability	5.92	3.04	7.92	0.92	
District boundary (shapefile)	-	-	-	-	Geological Survey of Bangladesh (2020)

## 4 Results

### 4.1 Sub-factors mapping and weighting

The spatial distribution of sub-factors is presented in Figure 2, and their respective values based on pairwise comparison are in the Table 3. The factors of spatial priority are in three broad categories: demographic, economic and vulnerability. Under the demographic category, five factors – the number of confirmed COVID-19 cases, percent of the total population, population density, percent of the urban people, and percent of the population over the age of 40 – are considered. Of these five factors, the highest eigenvalue (i.e., 0.66) was found for the ‘more than 600 confirmed COVID-19 cases per 100,000 population’ sub-factor.

**Table 3:** Pairwise comparison matrix, consistency ratio and weights of the sub-factor.

Factors	(1)	(2)	(3)	(4)	Eigenvalues
**Confirmed COVID-19 cases (per 100000 population)**					
(1) Less than 200	1				0.04
(2) 201-400	3	1			0.10
(3) 401-600	5	3	1		0.20
(4) More than 600	9	7	5	1	0.66
Consistency Ratio = 0.062802					
**Percent of total population**					
(1) Less than 1.00	1				0.08
(2) 1.01–2.00	2	1			0.13
(3) 2.01–3.00	3	2	1		0.23
(4) More than 3.00	5	5	3	1	0.56
Consistency Ratio = 0.021753					
**Population density (persons per square kilometers)**					
(1) Less than 500	1				0.06
(2) 501-1000	3	1			0.12
(3) 1001-1500	5	3	1		0.26
(4) More than 1500	9	4	3	1	0.56
Consistency Ratio = 0.031177					
**Percent of population over the age of 40**					
(1) Less than 23.00	1				0.10
(2) 23.01–25.00	2	1			0.16
(3) 25.01–27.00	3	2	1		0.27
(4) More than 27	4	3	2	1	0.47
Consistency Ratio = 0.011357					
**Percent of urban population**					
(1) Less than 15	1				0.07
(2) 15.01–20.00	2	1			0.14
(3) 20.01–25.00	5	2	1		0.29
(4) More than 25	7	3	2	1	0.50
Consistency Ratio = 0.00652					
**Percent of working-age people**					
(1) Less than 55.00	1				0.06
(2) 55.01–60.00	4	1			0.15
(3) 60.01–65.00	5	3	1		0.28
(4) More than 65	6	3	3	1	0.51
Consistency Ratio = 0.076849					
**Percent of industrial workers**					
(1) Less than 1.50	1				0.04
(2) 1.51–3.00	5	1			0.12
(3) 3.01–4.50	7	3	1		0.26
(4) More than 4.50	9	6	3	1	0.58
Consistency Ratio = 0.068109					
**Percent of the service sector employment**					
(1) Less than 7.00	1				0.05
(2) 7.01–9.00	4	1			0.15
(3) 9.01–11.00	5	2	1		0.24
(4) More than 11.00	7	4	3	1	0.56
Consistency Ratio = 0.038256					
**Percent of poor people**					
(1) Less than 20.00	1				0.07
(2) 20.01–30.00	3	1			0.16
(3) 30.01–40.00	4	2	1		0.28
(4) More than 40.00	5	4	2	1	0.49
Consistency Ratio = 0.026708					
**Percent of households with disability**					
(1) Less than 5.00	1				0.09
(2) 5.01–6.00	2	1			0.13
(3) 6.01–7.00	3	2	1		0.22
(4) More than 7.00	4	4	4	1	0.56
Consistency Ratio = 0.052439					

The demographic category hardly shows a spatial pattern except for the capital city Dhaka and its surrounding districts, and for Chittagong and Khulna districts (the second and third largest cities, respectively). Dhaka region, Chittagong and Khulna rank high up in the total number of confirmed COVID-19 cases. Expectedly, districts having the major cities clearly show a higher agglomeration of population and urban population than others. However, the population over the age of 40 shows a different priority as the younger population is located in urban and city areas. Therefore, spatial priority for vaccine rollout from the demographic perspective is not conclusive.

Secondly, the economic category consists of three factors: percent of service sector employment, percent of industrial employment and percent of working-age people. Among the employment category, industrial workers more than 4.50 percent, service sectors more than 11 percent, and working-age population more than 65 percent have the highest eigenvalues (0·58, 0·56 and 0·51, respectively). From Figure 2, Dhaka and Chittagong have spatial monopolies in the agglomeration of industrial and service sector employment. Expectedly, major cities like Dhaka and its surrounding districts, Chittagong, Khulna and Sylhet show a high concentration of service and industrial employment. However, there is no clear spatial pattern among the 64 districts when the working-age population and poverty distribution are considered.

The vulnerability category consists of two factors: percent of poor people and percent of households with disabilities. Households with disabilities more than 7 percent and the poor more than 40 percent have the highest eigenvalues (0.56 and 0.49, respectively) among the categories.

### 4.2 Factors weighting

The pairwise comparison matrix among the selected ten spatial variables is in Table 4. The number of confirmed COVID-19 cases and population density have the highest eigenvalues (0·31 and 0·20, respectively). Therefore, these two factors combined (eigenvalue 0·51) explain more than half of the spatial priority for vaccine rollout. The percent of the working-age people has the third-highest eigenvalue (0·11) followed by industrial employment (0·09), service sector workers (0·08) and urban population (0·07), respectively.

**Table 4:** Pairwise comparison matrix, consistency ratio and weighs of the factors.

Factors	(1)	(2)	(3)	(4)	(5)	(6)	(7)	(8)	(9)	(10)	Eigenvalues
**(1)** Confirmed COVID-19 cases	1										0.31
**(2)** Percent of total population	1/4	1									0.03
**(3)** Population density	1/3	5	1								0.20
**(4)** Percent of population over the age of 40	1/7	2	1/4	1							0.05
**(5)** Percent of urban population	1/8	5	1/5	3	1						0.07
**(6)** Percent of working-age people	1/5	3	1/3	2	3	1					0.11
**(7)** Percent of industrial workers	1/4	4	1/3	3	1	1/2	1				0.09
**(8)** Percent of the service sector employment	1/4	4	1/4	3	1	1/2	1	1			0.08
**(9)** Percentage of poor people	1/5	1/2	1/7	1/4	1/2	1/4	1/5	1/3	1		0.03
**(10)** Percent of households with disability	1/7	3	1/5	1/3	1/2	1/3	1/4	1/3	1	1	0.03
Consistency Ratio = 0.076259											

Percent of the poor, population over the age of 40, percent of total population and percent of households with disability – these four factors have the lowest weights (eigenvalue 0·05, 0·03, 0·03 and 0·03, respectively). Bangladesh government's current strategy is mass-vaccination for all over the age of 40 (recently updated to over 25 years). However, the pairwise comparison matrix shows that the population over the age of 40 has low priority in vaccination compared to the rest of the spatial factors.

### 4.3 Spatial overlay and priority districts

**Figure 3:**
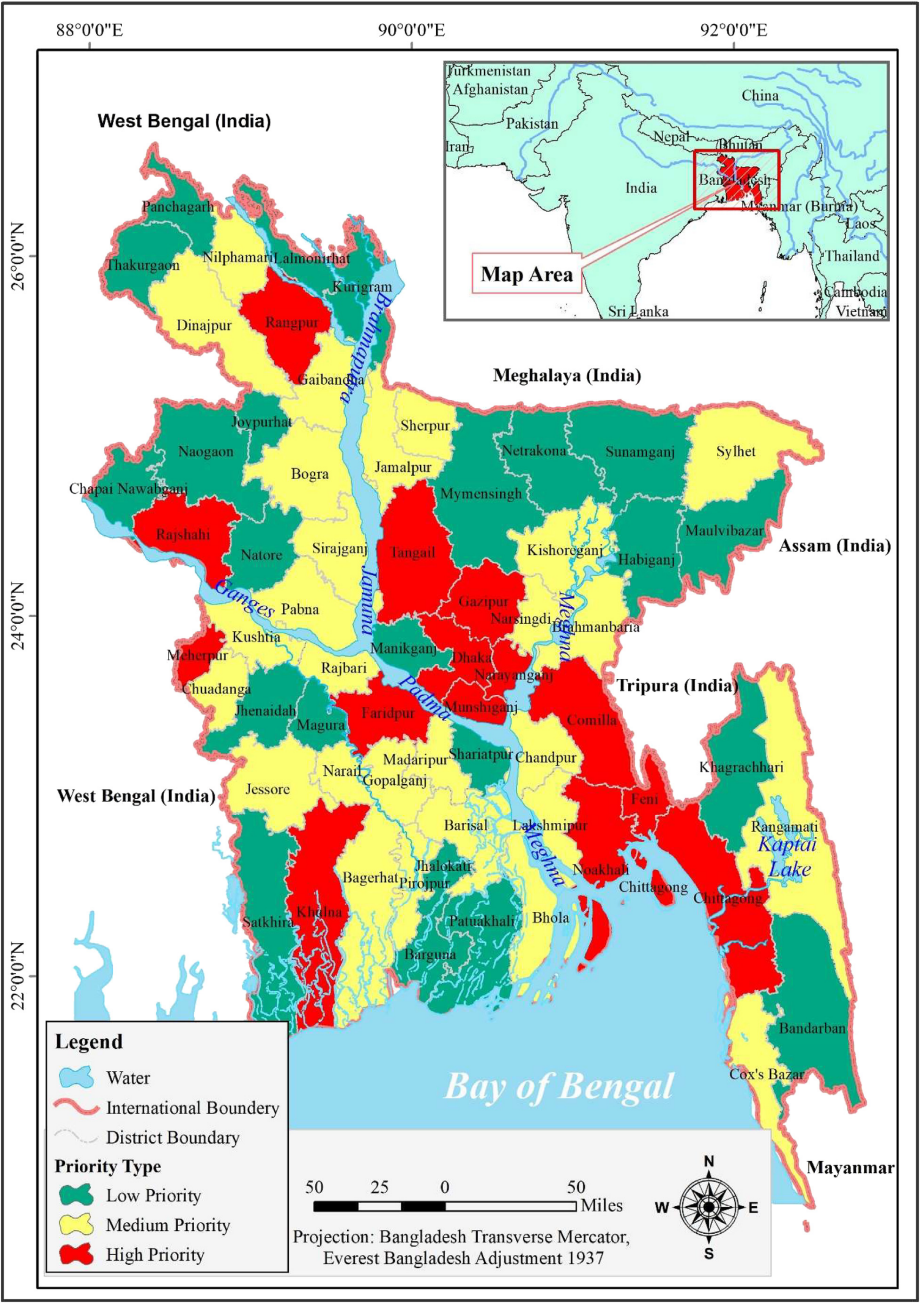
Spatial priority of 64 districts for vaccine rollout.

Priority indices of the individual sub-factors and factors using the spatial overlay are in Table 5. The priority indices are then categorized into three categories (i.e., high, medium and low priority districts for vaccine roll-out). The spatial distribution of priority districts are presented in Figure 3.

**Table 5:** Priority Index and Spatial Priority Index of 64 districts.

District Name	Priority Index	Priority Level	Spatial Priority Index	Spatial Priority Level	District Name	Priority Index	Priority Level	Spatial Priority Index	Spatial Priority Level
Dhaka	0.53082	High	0.643308	High	Dinajpur	0.163483	Medium	0.216454	Medium
Narayanganj	0.353231	High	0.461572	High	Rajbari	0.16212	Medium	0.264371	Medium
Chittagong	0.331456	High	0.380961	High	Madaripur	0.15961	Medium	0.276893	Low
Khulna	0.32721	High	0.417278	High	Rangamati	0.158353	Medium	0.198107	Low
Meherpur	0.32571	High	0.404698	High	Chuadanga	0.156537	Medium	0.240709	Low
Faridpur	0.322547	High	0.432961	High	Cox's Bazar	0.155463	Medium	0.176069	Low
Rajshahi	0.293057	High	0.36414	High	Bhola	0.155037	Medium	0.224889	Low
Rangpur	0.290958	High	0.351997	High	Bagerhat	0.153684	Medium	0.239346	Low
Munshiganj	0.28545	High	0.389385	High	Gopalganj	0.153037	Medium	0.252566	Low
Comilla	0.273631	High	0.3637	High	Lalmonirhat	0.144803	Low	0.191036	Low
Gazipur	0.269777	High	0.378119	High	Chapai Nawabganj	0.144515	Low	0.200241	Low
Feni	0.266634	High	0.340891	High	Panchagarh	0.141587	Low	0.176902	Low
Tangail	0.249647	High	0.350213	Medium	Jhalokati	0.138974	Low	0.226515	Low
Noakhali	0.248108	High	0.324828	Medium	Mymensingh	0.138095	Low	0.223498	Low
Bogra	0.231672	Medium	0.317982	Medium	Natore	0.13703	Low	0.22658	Low
Narsingdi	0.220499	Medium	0.322879	Medium	Joypurhat	0.135645	Low	0.207764	Low
Jamalpur	0.215843	Medium	0.287509	Medium	Bandarban	0.129836	Low	0.162818	Low
Chandpur	0.206429	Medium	0.299478	Medium	Sunamganj	0.129078	Low	0.159241	Low
Jessore	0.195266	Medium	0.294147	Medium	Thakurgaon	0.12842	Low	0.170215	Low
Sherpur	0.189497	Medium	0.25559	Medium	Shariatpur	0.125589	Low	0.224081	Low
Pabna	0.187436	Medium	0.277439	Medium	Magura	0.123619	Low	0.225351	Low
Brahmanbaria	0.184291	Medium	0.265546	Medium	Patuakhali	0.121881	Low	0.194973	Low
Pirojpur	0.179444	Medium	0.266014	Medium	Kurigram	0.121792	Low	0.167831	Low
Lakshmipur	0.17882	Medium	0.266944	Medium	Jhenaidah	0.120833	Low	0.218677	Low
Barisal	0.17863	Medium	0.264875	Medium	Satkhira	0.117416	Low	0.187916	Low
Kushtia	0.176827	Medium	0.280892	Medium	Netrakona	0.112779	Low	0.185222	Low
Kishoreganj	0.169399	Medium	0.252599	Medium	Naogaon	0.112141	Low	0.186334	Low
Narail	0.169071	Medium	0.265231	Medium	Barguna	0.110202	Low	0.167807	Low
Gaibandha	0.168646	Medium	0.233767	Medium	Khagrachhari	0.108035	Low	0.150283	Low
Nilphamari	0.166908	Medium	0.216769	Medium	Habiganj	0.103245	Low	0.167264	Low
Sirajganj	0.166516	Medium	0.259954	Medium	Manikganj	0.096881	Low	0.205223	Low
Sylhet	0.164131	Medium	0.207934	Medium	Maulvibazar	0.096653	Low	0.147648	Low

Figure 3 shows that Dhaka and its surrounding districts have a high priority for vaccine rollout. Similarly, Chittagong, Khulna and Rangpur districts – these divisional (2^nd^ tier) cities have high priority. Because the number of confirmed cases and population density are the two biggest factors (eigenvalue 0·31 and 0·20, respectively; in Table 4) for vaccine rollout priority, understandably, the divisional cities and their surrounding districts have the highest priority for vaccine rollout. Secondly, the medium priority districts are spatially clustered around the high priority districts whereas the low priority ones are scattered. Based on the weights of the ten factors, 14 districts were identified as high, 27 as medium and 23 as low priority for vaccine rollout.

**Figure 4:**
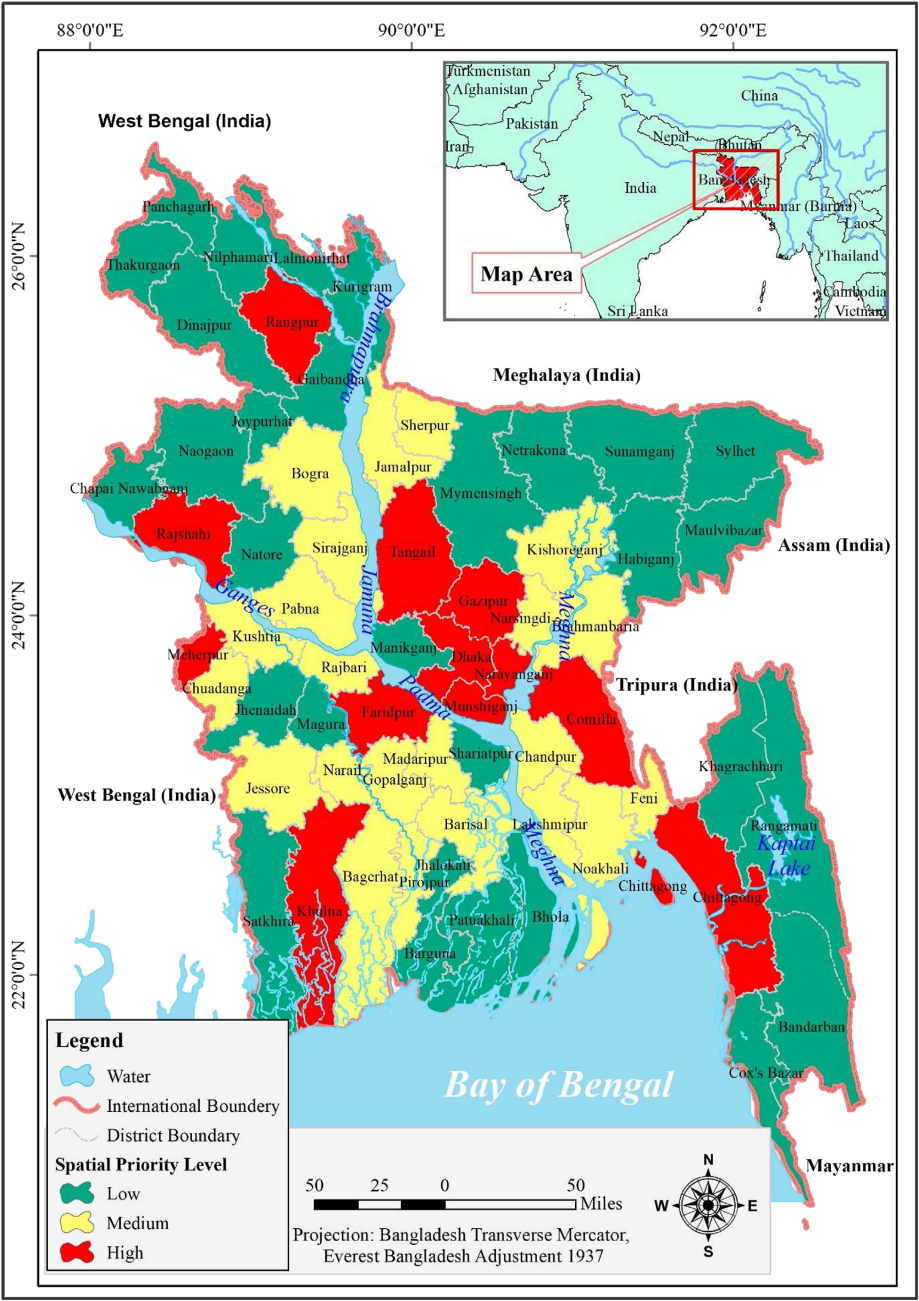
Proposed spatial priority of 64 districts for vaccine rollout.

Finally, the spatial priority of districts by incorporating distance function is presented in Figure 4, which has marked the high, medium and low priority districts for vaccine rollout. Out of the 64 districts, 12 are found as high priority, followed by 22 and 30 districts as medium and low priority, respectively.

## 5 Discussion

Mass vaccination is hardly a judicial strategy without ensuring vaccine availability. Therefore, the COVID-19 vaccine roll-out must be rationed and prioritized. The rollout strategy presented in this study is in line with the CDC and WHO guidelines. Four key factors i.e., demographic, economic, vulnerable groups and spatiality are considered for vaccine roll-out against limited supply. Among the vulnerable groups, the percent of households with disabilities and the poor are taken into consideration as the health workers and essential service providers are already been covered in the first phase of vaccination. Vaccine rollout strategies worldwide have overwhelmingly prioritized the descending age categories as they are the most vulnerable to COVID infection. Two major criticism to this mass-vaccination strategy is obvious. First, the supply of vaccines is limited against demand, and secondly, vaccine nationalization is an addition to the already strained supply chain. For example, India, the biggest producer of Oxford-AstraZeneca COVID-19 vaccine is prioritizing national demand over export. The economic cost of the pandemic is forecasted to aggravate due to unequal and limited supply of vaccines (see for example, [26]). Even after the third wave has leveled or even surpassed the first wave of the 2020s confirmed cases worldwide, a lockdown strategy is hardly an acceptable option due to economic and social costs, and lockdown fatigue. This, however, points to the importance of prioritizing the vulnerable groups, economic factors, and age-based vaccine rollout strategy.

Numerous studies link the number of COVID-19 cases to demographic factors, connectivity, exposed occupation, and urban poor and marginalized communities [11, 13, 14, 52]. Urban areas present a perfect combination of the aforementioned factors resulting in the soaring number of COVID-19 cases. However, urban areas contribute to more than 80% of the World's GDP, making it a priority for vaccine rollout. In the case of Bangladesh, urban areas present the largest concentration of industrial (i.e., ready-made garments) and service sector (i.e., transportation) employment of exposed nature, making vaccination the only way out for opening up economic activities and maintaining the functioning of the society.

Multi-criteria analysis using AHP of this study, therefore, accounts for the demographic, economic and vulnerability components to demonstrate spatial vaccine rollout strategy among 64 administrative districts in Bangladesh. The overlay of weighted sub-factors and factors identifies 14 high-priority districts for vaccine rollout (Figure 3). The 29 March 2021 press briefing by the Ministry of Health suggests that 29 of the 64 districts have the highest transmission trend in Bangladesh; 13 of which overlaps with the priority districts of this study. The findings of this study suggest that aside from the number of COVID-19 cases, population density (eigenvalue 0·20), percent of urban population (0·07), percent of the working-age population (0·11), and percent of industrial (0·09) and service sector employment (0·08) overwhelmingly indicate urban area a priority for vaccine rollout. This spatial distribution, however, is an indication that COVID-19 cases spread from urban centers to hinterland areas. Therefore, contrary to the finding of Boterman [12], our study shows a clear pattern of urbanization of cases, and thus, a priority for Bangladesh's national vaccination strategy.

In addition to the multi-criteria analysis for vaccine rollout, we consider the study districts spatially inter-connected. Therefore, the distance between the districts is inversely correlated with the COVID-19 transmission. We measured additional weight due to the inter-district distance factor (Figure 4). A total of 12 districts were identified as high-priority for vaccine rollout followed by 22 districts as medium priority. Similar to the multi-criteria analysis, the spatially high-priority districts comprise of the capital city Dhaka and the second-tier cities like Chittagong, Khulna, Rajshahi, and Rangpur. This, however, is an indication that urban population and population density are considerable factors for the vaccine roll-out strategy. Secondly, the priority districts include industrial and service center hubs like Narayanganj, Tangail and Munshiganj, meaning, the economic factors are accounted for in the spatial priority for vaccine roll-out.

The analysis presented in this study is at the district level due to a lack of data availability at a smaller scale. Several studies have highlighted the potential of cluster analysis for identifying the spatial distribution of COVID-19 cases at a small scale (see for example, [53, 54, 55]). Additionally, technological advancements like contract tracing applications have created new opportunities for identifying COVID-19 cases at a granular scale (see for example, [56, 57, 58]). GIS can play a critical role in mapping and visualizing the COVID-19 scenario at a spatial level. Thus, GIS is a visual aid in reaching a wider audience through cluster and spatial priority analysis, mapping and visualization in spatial policy regarding COVID-19. Despite the lack of granular scale analysis, this paper offers a sound methodological approach to spatial vaccine roll-out strategy against limited supply.

The Bangladesh government strategy has been mass-vaccination over the age of 40 (25 + as of 29 July 2021), which appears to be a mistake considering the uncertain and limited supply of vaccines. Therefore, it is pragmatic to have a national strategy that combines prolonged COVID-19 emergency preparedness, sustained economic activities, and reduced transmissibility against the shortage of vaccine supply. The data-driven spatial priority districts presented in this study can be a framework with ample opportunity for data addition and modification, and is replicable considering local and contextual priority for vaccine rollout strategy.

## 6 Conclusion

The COVID-19 vaccine shortage is to continue due to global high demand and limited supply. Bangladesh's mass-vaccination policy – currently, over the age of 25 – is sure to fall short of the COVID-19 vaccine supply. In a limited vaccine supply scenario, we have proposed an alternative spatial priority-based vaccine rollout strategy by weighing a combination of ten demographic, economic and vulnerability factors, and an additional spatial factor, for 64 districts of Bangladesh. The most significant factors of vaccine rollout strategy are the number of confirmed COVID-19 cases followed by population density, percent of the working-age people, percent of industrial and service sector employment, and percentage of the urban population. Based on the ten factors and a spatial distance factor, 12 districts were identified as high-priority, 22 as medium-priority and the rest as low-priority for vaccine rollout. A total of 9 of the 12 high-priority districts overlap with the findings of the Ministry of Health that marked 29 districts with the highest COVID-19 transmission trend. Our findings by no means recommend stopping mass vaccination but spatially prioritize vaccination against limited supply that can curb down COVID-19 transmission as well as keep the economy moving.

One of the major limitations of this study is the lack of granular scale analysis due to the lack of data availability. However, the methodological approach of this study can be applied on a small scale, and has ample opportunities for data aggregation while designing vaccine roll-out strategy according to any local context. Secondly, the study shows a spatial mapping of the COVID-19 factors weights as well as spatial connectivity as factors for virus transmission. This offers a practical method for creating a buffer within and between spatial units to curb COVID transmission through local suppressing measures. Finally, the proposed method can have broader application for spatial priority identification for suppressive and pro-active measures against other infectious and transmittable diseases like Tuberculosis and Dengue in Bangladesh and beyond.

## Declarations

### Author contribution statement

Showmitra Kumar Sarkar and Md. Manjur Morshed: Conceived and designed the experiments; Performed the experiments; Analyzed and interpreted the data; Contributed reagents, materials, analysis tools or data; Wrote the paper.

### Funding statement

This research did not receive any specific grant from funding agencies in the public, commercial, or not-for-profit sectors.

### Data availability statement

Data will be made available on request.

### Declaration of interests statement

The authors declare no conflict of interest.

### Additional information

No additional information is available for this paper.

## Footnotes

1 https://www.washingtonpost.com/world/2021/01/14/indonesia-coronavirus-vaccination-young/.

2 https://www.dw.com/en/bangladesh-india-coronavirus-vaccine/a-57200281.

3 https://www.dhakatribune.com/bangladesh/2021/07/18/bangladesh-receives-more-2-million-sinopharm-vaccine-doses-from-china.

4 https://www.economicsobservatory.com/why-has-coronavirus-affected-cities-more-rural-areas.

5 http://jedkolko.com/2020/06/21/the-changing-geography-of-covid19/.
